# Comparison of Integrated 3D Electroanatomic Mapping and Conventional Mapping Systems for Pentaspline Pulsed Field Ablation of Atrial Fibrillation

**DOI:** 10.1111/jce.70292

**Published:** 2026-03-02

**Authors:** Auroa Badin, Andrea K. Robinson, Allyson Farrah, Saanvi Billakanty, David M. Nemer, Ankur N. Shah, Jaret D. Tyler, Eugene Y. Fu, Nagesh Chopra, Anish K. Amin

**Affiliations:** ^1^ Division of Cardiac Electrophysiology, OhioHealth Heart and Vascular Physicians, Riverside Methodist Hospital Columbus Ohio USA; ^2^ Department of Immunity and Inflammation Cleveland Clinic Research Cleveland Ohio USA

**Keywords:** atrial fibrillation, cost analysis, electroanatomic mapping, procedural efficiency, pulmonary vein isolation, pulsed field ablation, radiation exposure, three‐dimensional mapping

## Abstract

**Introduction:**

Pulsed field ablation (PFA) is an established therapy for atrial fibrillation (AF), yet optimal mapping strategies during pentaspline PFA remain uncertain. We compared procedural characteristics and material costs of an integrated 3D electroanatomic mapping (EAM) platform with conventional mapping systems.

**Methods and Results:**

In a multicenter observational study of 448 consecutive AF ablations done using pentaspline catheter (62 integrated mapping; 386 conventional mapping) performed between February 2024 and August 2025, baseline characteristics were similar between groups. Compared with conventional mapping, the integrated platform was associated with shorter total procedure time (49 ± 16 vs. 66 ± 26 min, *p* < 0.001), shorter left atrial dwell time (35 ± 12 vs. 46 ± 30 min, *p* < 0.001), reduced fluoroscopy time (6 ± 4 vs. 12 ± 7 min, *p* < 0.001), lower radiation dose (36 ± 10 vs. 93 ± 18 mGy, *p* < 0.001), and lower mean material cost ($12 958 ± 164 vs. $14 739 ± 1192, *p* < 0.001). Differences were consistent across lesion sets. Acute pulmonary vein isolation was achieved in all patients, with two major complications occurring in the conventional mapping group.

**Conclusion:**

Integrated 3D EAM during pentaspline PFA was associated with shorter procedures, lower radiation exposure, and reduced material costs. These findings support the procedural and operational advantages of integrated mapping platforms in contemporary AF ablation practice.

Pulsed field ablation (PFA) was validated to be a safe and effective therapy for atrial fibrillation (AF) [1]. Compared to European workflows, US intraprocedural workflows rely heavily on general anesthesia, intracardiac ultrasound (ICE) guidance, and 3D electroanatomic mapping (EAM) [2]. Mapping while using the Pentaspline Farawave catheter (Boston Scientific) can be achieved by various mapping systems. Carto 3 (Biosense Webster) and Rhythmia HDX (Boston Scientific) require the use of an additional multielectrode catheter to create geometry, voltage map and to be able to track the pentaspline catheter, while Ensite system (Abbott) can visualize and create a map directly utilizing the pentaspline catheter. More recently, the Faraview Module allows the Opal HDx system (Boston Scientific) to visualize the size, shape, and rotation of the catheter, along collecting geometry and activation maps (Figure 1A).

**Figure 1:**
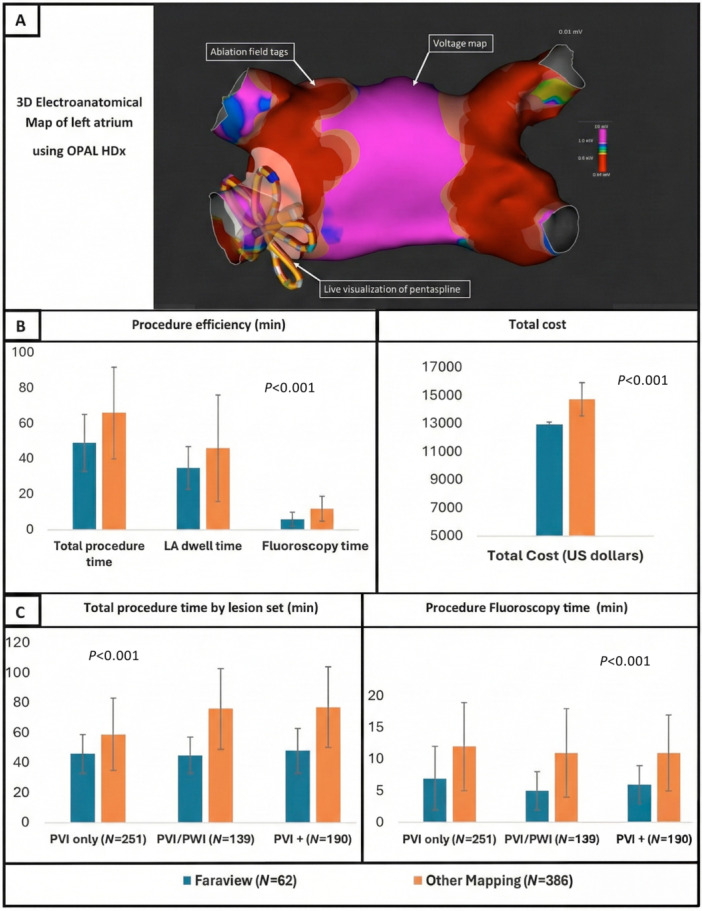
Faraview versus other mapping systems for pulsed field ablation of atrial fibrillation. (A) 3D Electroanatomical map of the left atrium using Opal HDx, (B) Procedure efficiency and total cost compared, (C) Total procedure and fluorsocpy time per lession set compared.

This analysis compared procedural workflow and material cost associated with the new Opal HDx Faraview catheter to other EAM mapping system at three high‐volume hospitals in central Ohio between February 2024 and August 2025. Twelve operators participated with varying pre‐commercial PFA experience. Operators completed ablation with user‐specified vascular access and diagnostic catheters. Intracardiac echo (ICE) was utilized in all cases consistent with standard of care in the United States. All procedures included pre‐ and post‐left atrium (LA) 3D map. Procedure characteristics, patient demographics, and total material costs of each case were analyzed based on the 3D EAM system used.

After excluding nonmapped cases, 448 patients were included: 62 were mapped with Faraview and 386 with other mapping systems. Baseline characteristics were similar between the groups: paroxysmal AF (48% vs. 57%), index ablation (68% vs. 74%), male (58% vs. 63%), age (69 vs. 68 years), BMI (33 vs. 32), LA volume index (32 vs. 34 mL/m^2^), respectively. Faraview procedures compared to other systems were shorter: total time (49 ± 16 vs. 66 ± 26 min, *p* < 0.001), LA dwell time (35 ± 12 vs. 46 ± 30 min, *p* < 0.001) and utilized less radiation: total fluoroscopy (6 ± 4 vs. 12 ± 7 min, *p* < 0.001), radiation dose (36 ± 10 vs. 93 ± 18 mGy, *p* < 0.001), with less average cost ($12 958 ± 164 vs. $14 739 ± 1192, *p* < 0.001), respectively (Figure 1B).

When analyzed by lesion set, these differences were consistent. For pulmonary vein isolation (PVI) only procedures, Faraview (*n* = 26) versus other mapping (*n* = 226) showed shorter total duration (46 ± 13 vs. 59 ± 24 min, *p* = 0.031), and fluoroscopy time (7 ± 5 vs. 12 ± 7 min, *p* < 0.001). Similarly, with PVI and posterior wall isolation (PWI), Faraview (*n* = 23) versus other mapping (*n* = 116): total duration (45 ± 12 vs. 76 ± 27 min, *p* < 0.001), fluoroscopy time (5 ± 3 vs. 11 ± 7 min, *p* < 0.001). Similar pattern was observed for PVI plus additional lesion sets, Faraview (*n* = 37) versus other mapping (*n* = 153): total duration (48 ± 15 vs. 77 ± 27 min, *p* < 0.001), fluoroscopy time (6 ± 3 vs. 11 ± 6 min, *p* < 0.001) (Figure 1C). Similarly, Faraview (62) total procedure time was shorter than Ensite HDx NAVx module with integrated Farapulse (154), but the difference was small: total time (49 ± 16 vs. 55.6 ± 20 min, *p* < 0.012), total fluoroscopy time (6 ± 4 vs. 13 ± 6 min, *p* < 0.001), respectively.

Acute PVI was achieved in all patients. Two major complications occurred: one intraperitoneal bleed and one access site pseudoaneurysm both occurred in the other mapping arm. Our analysis included patients with atypical macro atrial flutters (*n* = 16, 4 mapped with Faraview) that required activation mapping in which the Opal HDx system successfully guided ablation and termination of flutter in all 4 cases.

These findings are consistent with procedural efficiency expected from integrated mapping platform, which negates the need to exchange the pentaspline PFA catheter to another multielectrode catheter pre‐ and post‐ablation, thereby saving on time, fluoroscopy, and overall cost.

The observation that PVI‐only and PVI + PWI procedures demonstrated similar procedural durations in the Faraview cohort warrants consideration. PWI using the pentaspline PFA catheter typically requires a limited number of additional applications, often adding minimal incremental time compared with PVI alone. In addition, the relatively small number of cases in these subgroups limits the ability to detect modest differences in procedural metrics. Operator experience and case selection may also have contributed, with early adopters of the platform more likely to perform PVI‐only procedures. In contrast, alternative mapping workflows often require additional remapping and catheter exchanges prior to PWI, which may disproportionately increase procedure duration.

As previously demonstrated, workflow can significantly impact procedural efficiency and cost of care [2, 3]. The value of 3D EAM in PFA is still debated with some reports showing no difference in outcomes [3] and other favoring its use to reduce fluoroscopy time [4]. This evaluation demonstrates that integrated 3D EAM in the new Faraview module was more efficient and cost‐saving when compared to using other mapping systems. Furthermore, in addition to procedural efficiency, the use of a single catheter for both mapping and ablation may reduce the risk of silent cerebral events (SCE), which have been shown to occur more frequently with multiple catheter exchanges in the LA (40% vs. 13%) [5]. It is plausible that those SCE may even be higher when exchanging catheters through the larger sheath (16 Fr) required for the Farawave catheter.

While we strongly believe this observational study provides valuable real‐world evidence, it is limited by its observational design, lack of randomization, and absence of long‐term clinical outcomes such as arrhythmia recurrence. Randomized trials with long‐term follow‐up are essential to establish the effectiveness of integrated mapping platforms. Our findings provide a strong foundation for clinical decision‐making and technology adaptation in routine practice. As healthcare systems face increasing pressure to optimize resource utilization, while maintaining quality outcomes, technologies that improve efficiency, safety, and cost effectiveness and that may reduce the risk of air embolization, provide substantial clinical and operational value.

## Funding

The authors received no specific funding for this work.

## Ethics Statement

This study protocol was approved by OhioHealth IRB.

## Consent

Patient consent was waived.

## Conflicts of Interest

Dr. Badin has served as a consultant for Biosense Webster and Boston Scientific. Dr. Amin has served as a consultant for Biosense Webster, Abbott, and Boston Scientific.
